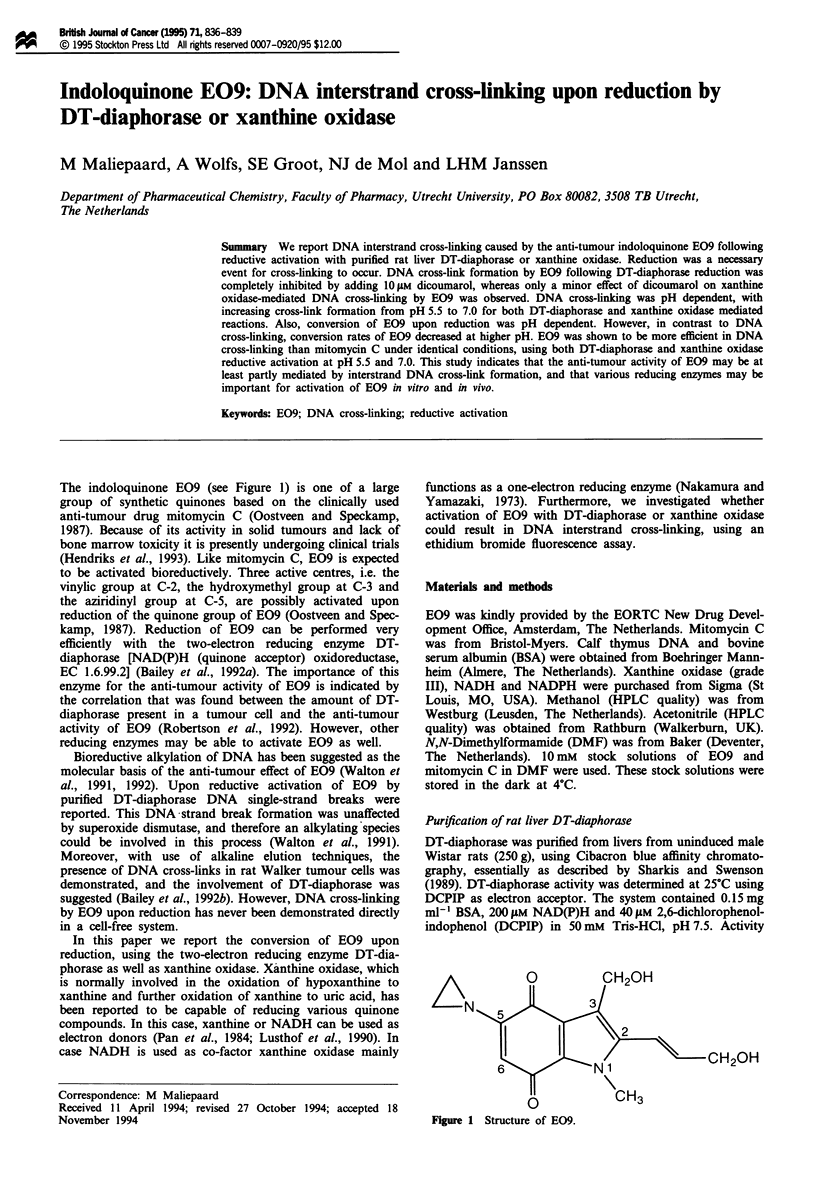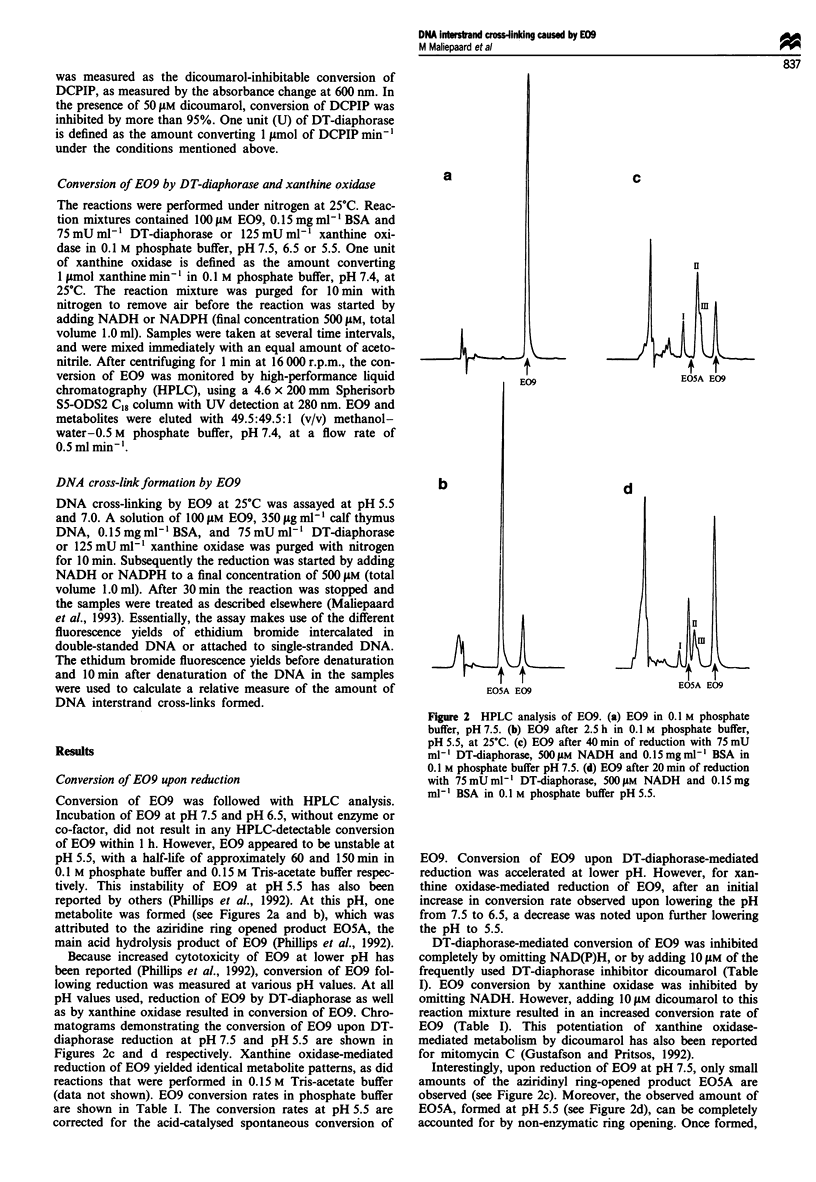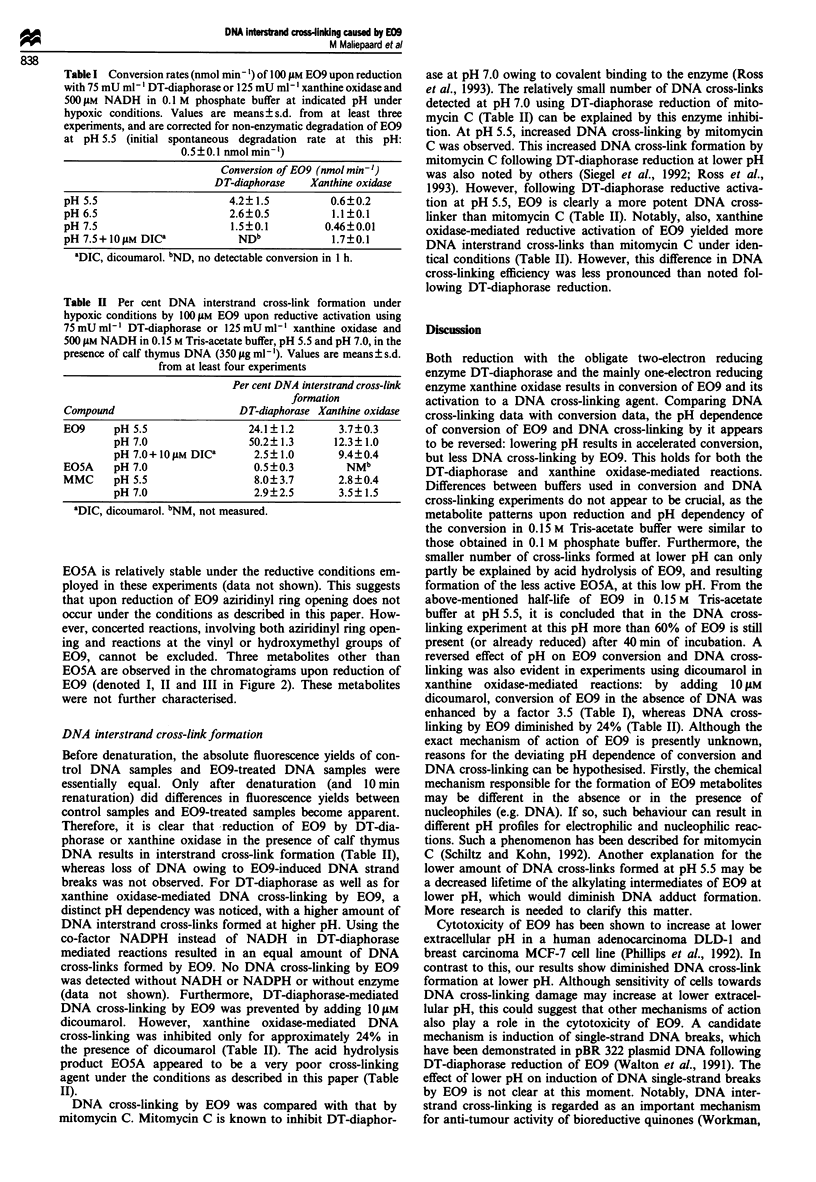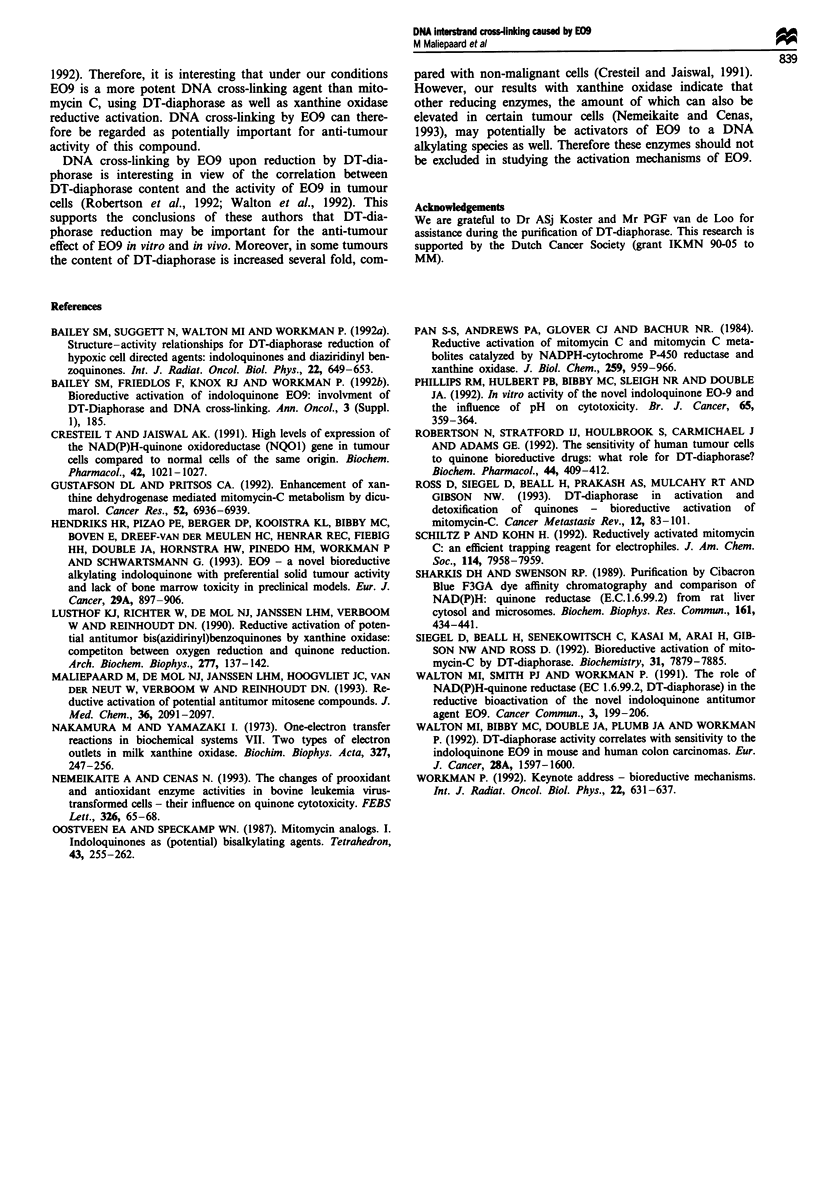# Indoloquinone EO9: DNA interstrand cross-linking upon reduction by DT-diaphorase or xanthine oxidase.

**DOI:** 10.1038/bjc.1995.161

**Published:** 1995-04

**Authors:** M. Maliepaard, A. Wolfs, S. E. Groot, N. J. de Mol, L. H. Janssen

**Affiliations:** Department of Pharmaceutical Chemistry, Faculty of Pharmacy, Utrecht University, The Netherlands.

## Abstract

We report DNA interstrand cross-linking caused by the anti-tumour indoloquinone EO9 following reductive activation with purified rat liver DT-diaphorase or xanthine oxidase. Reduction was a necessary event for cross-linking to occur. DNA cross-link formation by EO9 following DT-diaphorase reduction was completely inhibited by addition 10 microM dicoumarol, whereas only a minor effect of dicoumarol on xanthine oxidase-mediated DNA cross-linking by EO9 was observed. DNA cross-linking was pH dependent, with increasing cross-link formation from pH 5.5 to 7.0 for both DT-diaphorase and xanthine oxidase mediated reactions. Also, conversion of EO9 upon reduction was pH dependent. However, in contrast to DNA cross-linking, conversion rates of EO9 decreased at higher pH. EO9 was shown to be more efficient in DNA cross-linking than mitomycin C under identical conditions, using both DT-diaphorase and xanthine oxidase reductive activation at pH 5.5 and 7.0. This study indicates that the anti-tumour activity of EO9 may be at least partly mediated by interstrand DNA cross-link formation, and that various reducing enzymes may be important for activation of EO9 in vitro and in vivo.


British Journal of Cancer (1995) 71, 836-839
© 1995 Stockton Press Ltd All rights reserved 0007-0920/95 $12.00

### Indoloquinone EO9: DNA interstrand cross-linking upon reduction by DT-diaphorase or xanthine oxidase

M Maliepaard, A Wolfs, SE Groot, NJ de Mol and LHM Janssen

*Department of Pharmaceutical Chemistry, Faculty of Pharmacy, Utrecht University, PO Box 80082, 3508 TB Utrecht, The Netherlands*

**Summary** We report DNA interstrand cross-linking caused by the anti-tumour indoloquinone EO9 following reductive activation with purified rat liver DT-diaphorase or xanthine oxidase. Reduction was a necessary event for cross-linking to occur. DNA cross-link formation by EO9 following DT-diaphorase reduction was completely inhibited by adding 10 μM dicoumarol, whereas only a minor effect of dicoumarol on xanthine oxidase-mediated DNA cross-linking by EO9 was observed. DNA cross-linking was pH dependent, with increasing cross-link formation from pH 5.5 to 7.0 for both DT-diaphorase and xanthine oxidase mediated reactions. Also, conversion of EO9 upon reduction was pH dependent. However, in contrast to DNA cross-linking, conversion rates of EO9 decreased at higher pH. EO9 was shown to be more efficient in DNA cross-linking than mitomycin C under identical conditions, using both DT-diaphorase and xanthine oxidase reductive activation at pH 5.5 and 7.0. This study indicates that the anti-tumour activity of EO9 may be at least partly mediated by interstrand DNA cross-link formation, and that various reducing enzymes may be important for activation of EO9 *in vitro* and *in vivo*.

**Keywords:** EO9; DNA cross-linking; reductive activation

The indoloquinone EO9 (see Figure 1) is one of a large group of synthetic quinones based on the clinically used anti-tumour drug mitomycin C (Oostveen and Speckamp, 1987). Because of its activity in solid tumours and lack of bone marrow toxicity it is presently undergoing clinical trials (Hendriks *et al.*, 1993). Like mitomycin C, EO9 is expected to be activated bioreductively. Three active centres, i.e. the vinylic group at C-2, the hydroxymethyl group at C-3 and the aziridinyl group at C-5, are possibly activated upon reduction of the quinone group of EO9 (Oostveen and Speckamp, 1987). Reduction of EO9 can be performed very efficiently with the two-electron reducing enzyme DT-diaphorase [NAD(P)H (quinone acceptor) oxidoreductase, EC 1.6.99.2] (Bailey *et al.*, 1992*a*). The importance of this enzyme for the anti-tumour activity of EO9 is indicated by the correlation that was found between the amount of DT-diaphorase present in a tumour cell and the anti-tumour activity of EO9 (Robertson *et al.*, 1992). However, other reducing enzymes may be able to activate EO9 as well.

Bioreductive alkylation of DNA has been suggested as the molecular basis of the anti-tumour effect of EO9 (Walton *et al.*, 1991, 1992). Upon reductive activation of EO9 by purified DT-diaphorase DNA single-strand breaks were reported. This DNA strand break formation was unaffected by superoxide dismutase, and therefore an alkylating species could be involved in this process (Walton *et al.*, 1991). Moreover, with use of alkaline elution techniques, the presence of DNA cross-links in rat Walker tumour cells was demonstrated, and the involvement of DT-diaphorase was suggested (Bailey *et al.*, 1992*b*). However, DNA cross-linking by EO9 upon reduction has never been demonstrated directly in a cell-free system.

In this paper we report the conversion of EO9 upon reduction, using the two-electron reducing enzyme DT-diaphorase as well as xanthine oxidase. Xanthine oxidase, which is normally involved in the oxidation of hypoxanthine to xanthine and further oxidation of xanthine to uric acid, has been reported to be capable of reducing various quinone compounds. In this case, xanthine or NADH can be used as electron donors (Pan *et al.*, 1984; Lusthof *et al.*, 1990). In case NADH is used as co-factor xanthine oxidase mainly functions as a one-electron reducing enzyme (Nakamura and Yamazaki, 1973). Furthermore, we investigated whether activation of EO9 with DT-diaphorase or xanthine oxidase could result in DNA interstrand cross-linking, using an ethidium bromide fluorescence assay.

### Materials and methods

EO9 was kindly provided by the EORTC New Drug Development Office, Amsterdam, The Netherlands. Mitomycin C was from Bristol-Myers. Calf thymus DNA and bovine serum albumin (BSA) were obtained from Boehringer Mannheim (Almere, The Netherlands). Xanthine oxidase (grade III), NADH and NADPH were purchased from Sigma (St Louis, MO, USA). Methanol (HPLC quality) was from Westburg (Leusden, The Netherlands). Acetonitrile (HPLC quality) was obtained from Rathburn (Walkerburn, UK). *N,N*-Dimethylformamide (DMF) was from Baker (Deventer, The Netherlands). 10 mM stock solutions of EO9 and mitomycin C in DMF were used. These stock solutions were stored in the dark at 4°C.

### *Purification of rat liver DT-diaphorase*

DT-diaphorase was purified from livers from uninduced male Wistar rats (250 g), using Cibacron blue affinity chromatography, essentially as described by Sharkis and Swenson (1989). DT-diaphorase activity was determined at 25°C using DCPIP as electron acceptor. The system contained 0.15 mg $ml^{-1}$ BSA, 200 μM NAD(P)H and 40 μM 2,6-dichlorophenol-indophenol (DCPIP) in 50 mM Tris-HCl, pH 7.5. Activity

**Figure 1** Structure of EO9.

Correspondence: M Maliepaard
Received 11 April 1994; revised 27 October 1994; accepted 18 November 1994

was measured as the dicoumarol-inhibitable conversion of DCPIP, as measured by the absorbance change at 600 nm. In the presence of 50 μM dicoumarol, conversion of DCPIP was inhibited by more than 95%. One unit (U) of DT-diaphorase is defined as the amount converting 1 μmol of DCPIP $min^{-1}$ under the conditions mentioned above.

*Conversion of EO9 by DT-diaphorase and xanthine oxidase*

The reactions were performed under nitrogen at 25°C. Reaction mixtures contained 100 μM EO9, 0.15 mg $ml^{-1}$ BSA and 75 mU $ml^{-1}$ DT-diaphorase or 125 mU $ml^{-1}$ xanthine oxidase in 0.1 M phosphate buffer, pH 7.5, 6.5 or 5.5. One unit of xanthine oxidase is defined as the amount converting 1 μmol xanthine $min^{-1}$ in 0.1 M phosphate buffer, pH 7.4, at 25°C. The reaction mixture was purged for 10 min with nitrogen to remove air before the reaction was started by adding NADH or NADPH (final concentration 500 μM, total volume 1.0 ml). Samples were taken at several time intervals, and were mixed immediately with an equal amount of acetonitrile. After centrifuging for 1 min at 16 000 r.p.m., the conversion of EO9 was monitored by high-performance liquid chromatography (HPLC), using a 4.6 × 200 mm Spherisorb S5-ODS2 $C_{18}$ column with UV detection at 280 nm. EO9 and metabolites were eluted with 49.5:49.5:1 (v/v) methanol–water–0.5 M phosphate buffer, pH 7.4, at a flow rate of 0.5 ml $min^{-1}$.

*DNA cross-link formation by EO9*

DNA cross-linking by EO9 at 25°C was assayed at pH 5.5 and 7.0. A solution of 100 μM EO9, 350 μg $ml^{-1}$ calf thymus DNA, 0.15 mg $ml^{-1}$ BSA, and 75 mU $ml^{-1}$ DT-diaphorase or 125 mU $ml^{-1}$ xanthine oxidase was purged with nitrogen for 10 min. Subsequently the reduction was started by adding NADH or NADPH to a final concentration of 500 μM (total volume 1.0 ml). After 30 min the reaction was stopped and the samples were treated as described elsewhere (Maliepaard *et al.*, 1993). Essentially, the assay makes use of the different fluorescence yields of ethidium bromide intercalated in double-standed DNA or attached to single-stranded DNA. The ethidum bromide fluorescence yields before denaturation and 10 min after denaturation of the DNA in the samples were used to calculate a relative measure of the amount of DNA interstrand cross-links formed.

### Results

*Conversion of EO9 upon reduction*

Conversion of EO9 was followed with HPLC analysis. Incubation of EO9 at pH 7.5 and pH 6.5, without enzyme or co-factor, did not result in any HPLC-detectable conversion of EO9 within 1 h. However, EO9 appeared to be unstable at pH 5.5, with a half-life of approximately 60 and 150 min in 0.1 M phosphate buffer and 0.15 M Tris-acetate buffer respectively. This instability of EO9 at pH 5.5 has also been reported by others (Phillips *et al.*, 1992). At this pH, one metabolite was formed (see Figures 2a and b), which was attributed to the aziridine ring opened product EO5A, the main acid hydrolysis product of EO9 (Phillips *et al.*, 1992).

Because increased cytotoxicity of EO9 at lower pH has been reported (Phillips *et al.*, 1992), conversion of EO9 following reduction was measured at various pH values. At all pH values used, reduction of EO9 by DT-diaphorase as well as by xanthine oxidase resulted in conversion of EO9. Chromatograms demonstrating the conversion of EO9 upon DT-diaphorase reduction at pH 7.5 and pH 5.5 are shown in Figures 2c and d respectively. Xanthine oxidase-mediated reduction of EO9 yielded identical metabolite patterns, as did reactions that were performed in 0.15 M Tris-acetate buffer (data not shown). EO9 conversion rates in phosphate buffer are shown in Table I. The conversion rates at pH 5.5 are corrected for the acid-catalysed spontaneous conversion of

**Figure 2** HPLC analysis of EO9. (**a**) EO9 in 0.1 M phosphate buffer, pH 7.5. (**b**) EO9 after 2.5 h in 0.1 M phosphate buffer, pH 5.5, at 25°C. (**c**) EO9 after 40 min of reduction with 75 mU $ml^{-1}$ DT-diaphorase, 500 μM NADH and 0.15 mg $ml^{-1}$ BSA in 0.1 M phosphate buffer pH 7.5. (**d**) EO9 after 20 min of reduction with 75 mU $ml^{-1}$ DT-diaphorase, 500 μM NADH and 0.15 mg $ml^{-1}$ BSA in 0.1 M phosphate buffer pH 5.5.

EO9. Conversion of EO9 upon DT-diaphorase-mediated reduction was accelerated at lower pH. However, for xanthine oxidase-mediated reduction of EO9, after an initial increase in conversion rate observed upon lowering the pH from 7.5 to 6.5, a decrease was noted upon further lowering the pH to 5.5.

DT-diaphorase-mediated conversion of EO9 was inhibited completely by omitting NAD(P)H, or by adding 10 μM of the frequently used DT-diaphorase inhibitor dicoumarol (Table I). EO9 conversion by xanthine oxidase was inhibited by omitting NADH. However, adding 10 μM dicoumarol to this reaction mixture resulted in an increased conversion rate of EO9 (Table I). This potentiation of xanthine oxidase-mediated metabolism by dicoumarol has also been reported for mitomycin C (Gustafson and Pritsos, 1992).

Interestingly, upon reduction of EO9 at pH 7.5, only small amounts of the aziridinyl ring-opened product EO5A are observed (see Figure 2c). Moreover, the observed amount of EO5A, formed at pH 5.5 (see Figure 2d), can be completely accounted for by non-enzymatic ring opening. Once formed,

**Table I** Conversion rates (nmol $min^{-1}$) of 100 μM EO9 upon reduction with 75 mU $ml^{-1}$ DT-diaphorase or 125 mU $ml^{-1}$ xanthine oxidase and 500 μM NADH in 0.1 M phosphate buffer at indicated pH under hypoxic conditions. Values are means ± s.d. from at least three experiments, and are corrected for non-enzymatic degradation of EO9 at pH 5.5 (initial spontaneous degradation rate at this pH: 0.5 ± 0.1 nmol $min^{-1}$)

| | *Conversion of EO9 (nmol min⁻¹)* | |
|---|---|---|
| | *DT-diaphorase* | *Xanthine oxidase* |
| pH 5.5 | 4.2 ± 1.5 | 0.6 ± 0.2 |
| pH 6.5 | 2.6 ± 0.5 | 1.1 ± 0.1 |
| pH 7.5 | 1.5 ± 0.1 | 0.46 ± 0.01 |
| pH 7.5 + 10 μM DIC[a] | ND[b] | 1.7 ± 0.1 |

[a]DIC, dicoumarol. [b]ND, no detectable conversion in 1 h.

**Table II** Per cent DNA interstrand cross-link formation under hypoxic conditions by 100 μM EO9 upon reductive activation using 75 mU $ml^{-1}$ DT-diaphorase or 125 mU $ml^{-1}$ xanthine oxidase and 500 μM NADH in 0.15 M Tris-acetate buffer, pH 5.5 and pH 7.0, in the presence of calf thymus DNA (350 μg $ml^{-1}$). Values are means ± s.d. from at least four experiments

| | | *Per cent DNA interstrand cross-link formation* | |
|---|---|---|---|
| *Compound* | | *DT-diaphorase* | *Xanthine oxidase* |
| EO9 | pH 5.5 | 24.1 ± 1.2 | 3.7 ± 0.3 |
| | pH 7.0 | 50.2 ± 1.3 | 12.3 ± 1.0 |
| | pH 7.0 + 10 μM DIC[a] | 2.5 ± 1.0 | 9.4 ± 0.4 |
| EO5A | pH 7.0 | 0.5 ± 0.3 | NM[b] |
| MMC | pH 5.5 | 8.0 ± 3.7 | 2.8 ± 0.4 |
| | pH 7.0 | 2.9 ± 2.5 | 3.5 ± 1.5 |

[a]DIC, dicoumarol. [b]NM, not measured.

EO5A is relatively stable under the reductive conditions employed in these experiments (data not shown). This suggests that upon reduction of EO9 aziridinyl ring opening does not occur under the conditions as described in this paper. However, concerted reactions, involving both aziridinyl ring opening and reactions at the vinyl or hydroxymethyl groups of EO9, cannot be excluded. Three metabolites other than EO5A are observed in the chromatograms upon reduction of EO9 (denoted I, II and III in Figure 2). These metabolites were not further characterised.

### *DNA interstrand cross-link formation*

Before denaturation, the absolute fluorescence yields of control DNA samples and EO9-treated DNA samples were essentially equal. Only after denaturation (and 10 min renaturation) did differences in fluorescence yields between control samples and EO9-treated samples become apparent. Therefore, it is clear that reduction of EO9 by DT-diaphorase or xanthine oxidase in the presence of calf thymus DNA results in interstrand cross-link formation (Table II), whereas loss of DNA owing to EO9-induced DNA strand breaks was not observed. For DT-diaphorase as well as for xanthine oxidase-mediated DNA cross-linking by EO9, a distinct pH dependency was noticed, with a higher amount of DNA interstrand cross-links formed at higher pH. Using the co-factor NADPH instead of NADH in DT-diaphorase mediated reactions resulted in an equal amount of DNA cross-links formed by EO9. No DNA cross-linking by EO9 was detected without NADH or NADPH or without enzyme (data not shown). Furthermore, DT-diaphorase-mediated DNA cross-linking by EO9 was prevented by adding 10 μM dicoumarol. However, xanthine oxidase-mediated DNA cross-linking was inhibited only for approximately 24% in the presence of dicoumarol (Table II). The acid hydrolysis product EO5A appeared to be a very poor cross-linking agent under the conditions as described in this paper (Table II).

DNA cross-linking by EO9 was compared with that by mitomycin C. Mitomycin C is known to inhibit DT-diaphorase at pH 7.0 owing to covalent binding to the enzyme (Ross *et al.*, 1993). The relatively small number of DNA cross-links detected at pH 7.0 using DT-diaphorase reduction of mitomycin C (Table II) can be explained by this enzyme inhibition. At pH 5.5, increased DNA cross-linking by mitomycin C was observed. This increased DNA cross-link formation by mitomycin C following DT-diaphorase reduction at lower pH was also noted by others (Siegel *et al.*, 1992; Ross *et al.*, 1993). However, following DT-diaphorase reductive activation at pH 5.5, EO9 is clearly a more potent DNA cross-linker than mitomycin C (Table II). Notably, also, xanthine oxidase-mediated reductive activation of EO9 yielded more DNA interstrand cross-links than mitomycin C under identical conditions (Table II). However, this difference in DNA cross-linking efficiency was less pronounced than noted following DT-diaphorase reduction.

### Discussion

Both reduction with the obligate two-electron reducing enzyme DT-diaphorase and the mainly one-electron reducing enzyme xanthine oxidase results in conversion of EO9 and its activation to a DNA cross-linking agent. Comparing DNA cross-linking data with conversion data, the pH dependence of conversion of EO9 and DNA cross-linking by it appears to be reversed: lowering pH results in accelerated conversion, but less DNA cross-linking by EO9. This holds for both the DT-diaphorase and xanthine oxidase-mediated reactions. Differences between buffers used in conversion and DNA cross-linking experiments do not appear to be crucial, as the metabolite patterns upon reduction and pH dependency of the conversion in 0.15 M Tris-acetate buffer were similar to those obtained in 0.1 M phosphate buffer. Furthermore, the smaller number of cross-links formed at lower pH can only partly be explained by acid hydrolysis of EO9, and resulting formation of the less active EO5A, at this low pH. From the above-mentioned half-life of EO9 in 0.15 M Tris-acetate buffer at pH 5.5, it is concluded that in the DNA cross-linking experiment at this pH more than 60% of EO9 is still present (or already reduced) after 40 min of incubation. A reversed effect of pH on EO9 conversion and DNA cross-linking was also evident in experiments using dicoumarol in xanthine oxidase-mediated reactions: by adding 10 μM dicoumarol, conversion of EO9 in the absence of DNA was enhanced by a factor 3.5 (Table I), whereas DNA cross-linking by EO9 diminished by 24% (Table II). Although the exact mechanism of action of EO9 is presently unknown, reasons for the deviating pH dependence of conversion and DNA cross-linking can be hypothesised. Firstly, the chemical mechanism responsible for the formation of EO9 metabolites may be different in the absence or in the presence of nucleophiles (e.g. DNA). If so, such behaviour can result in different pH profiles for electrophilic and nucleophilic reactions. Such a phenomenon has been described for mitomycin C (Schiltz and Kohn, 1992). Another explanation for the lower amount of DNA cross-links formed at pH 5.5 may be a decreased lifetime of the alkylating intermediates of EO9 at lower pH, which would diminish DNA adduct formation. More research is needed to clarify this matter.

Cytotoxicity of EO9 has been shown to increase at lower extracellular pH in a human adenocarcinoma DLD-1 and breast carcinoma MCF-7 cell line (Phillips *et al.*, 1992). In contrast to this, our results show diminished DNA cross-link formation at lower pH. Although sensitivity of cells towards DNA cross-linking damage may increase at lower extracellular pH, this could suggest that other mechanisms of action also play a role in the cytotoxicity of EO9. A candidate mechanism is induction of single-strand DNA breaks, which have been demonstrated in pBR 322 plasmid DNA following DT-diaphorase reduction of EO9 (Walton *et al.*, 1991). The effect of lower pH on induction of DNA single-strand breaks by EO9 is not clear at this moment. Notably, DNA interstrand cross-linking is regarded as an important mechanism for anti-tumour activity of bioreductive quinones (Workman,

1992). Therefore, it is interesting that under our conditions EO9 is a more potent DNA cross-linking agent than mitomycin C, using DT-diaphorase as well as xanthine oxidase reductive activation. DNA cross-linking by EO9 can therefore be regarded as potentially important for anti-tumour activity of this compound.

DNA cross-linking by EO9 upon reduction by DT-diaphorase is interesting in view of the correlation between DT-diaphorase content and the activity of EO9 in tumour cells (Robertson *et al.*, 1992; Walton *et al.*, 1992). This supports the conclusions of these authors that DT-diaphorase reduction may be important for the anti-tumour effect of EO9 *in vitro* and *in vivo*. Moreover, in some tumours the content of DT-diaphorase is increased several fold, compared with non-malignant cells (Cresteil and Jaiswal, 1991). However, our results with xanthine oxidase indicate that other reducing enzymes, the amount of which can also be elevated in certain tumour cells (Nemeikaite and Cenas, 1993), may potentially be activators of EO9 to a DNA alkylating species as well. Therefore these enzymes should not be excluded in studying the activation mechanisms of EO9.

**Acknowledgements**

We are grateful to Dr ASj Koster and Mr PGF van de Loo for assistance during the purification of DT-diaphorase. This research is supported by the Dutch Cancer Society (grant IKMN 90-05 to MM).

**References**

BAILEY SM, SUGGETT N, WALTON MI AND WORKMAN P. (1992*a*). Structure–activity relationships for DT-diaphorase reduction of hypoxic cell directed agents: indoloquinones and diaziridinyl benzoquinones. *Int. J. Radiat. Oncol. Biol. Phys.*, **22**, 649–653.

BAILEY SM, FRIEDLOS F, KNOX RJ AND WORKMAN P. (1992*b*). Bioreductive activation of indoloquinone EO9: involvment of DT-Diaphorase and DNA cross-linking. *Ann. Oncol.*, 3 (Suppl. 1), 185.

CRESTEIL T AND JAISWAL AK. (1991). High levels of expression of the NAD(P)H-quinone oxidoreductase (NQO1) gene in tumour cells compared to normal cells of the same origin. *Biochem. Pharmacol.*, **42**, 1021–1027.

GUSTAFSON DL AND PRITSOS CA. (1992). Enhancement of xanthine dehydrogenase mediated mitomycin-C metabolism by dicumarol. *Cancer Res.*, **52**, 6936–6939.

HENDRIKS HR, PIZAO PE, BERGER DP, KOOISTRA KL, BIBBY MC, BOVEN E, DREEF-VAN DER MEULEN HC, HENRAR REC, FIEBIG HH, DOUBLE JA, HORNSTRA HW, PINEDO HM, WORKMAN P AND SCHWARTSMANN G. (1993). EO9 – a novel bioreductive alkylating indoloquinone with preferential solid tumour activity and lack of bone marrow toxicity in preclinical models. *Eur. J. Cancer*, **29A**, 897–906.

LUSTHOF KJ, RICHTER W, DE MOL NJ, JANSSEN LHM, VERBOOM W AND REINHOUDT DN. (1990). Reductive activation of potential antitumor bis(aziridinyl)benzoquinones by xanthine oxidase: competiton between oxygen reduction and quinone reduction. *Arch. Biochem. Biophys.*, **277**, 137–142.

MALIEPAARD M, DE MOL NJ, JANSSEN LHM, HOOGVLIET JC, VAN DER NEUT W, VERBOOM W AND REINHOUDT DN. (1993). Reductive activation of potential antitumor mitosene compounds. *J. Med. Chem.*, **36**, 2091–2097.

NAKAMURA M AND YAMAZAKI I. (1973). One-electron transfer reactions in biochemical systems VII. Two types of electron outlets in milk xanthine oxidase. *Biochim. Biophys. Acta*, **327**, 247–256.

NEMEIKAITE A AND CENAS N. (1993). The changes of prooxidant and antioxidant enzyme activities in bovine leukemia virus-transformed cells – their influence on quinone cytotoxicity. *FEBS Lett.*, **326**, 65–68.

OOSTVEEN EA AND SPECKAMP WN. (1987). Mitomycin analogs. I. Indoloquinones as (potential) bisalkylating agents. *Tetrahedron*, **43**, 255–262.

PAN S-S, ANDREWS PA, GLOVER CJ AND BACHUR NR. (1984). Reductive activation of mitomycin C and mitomycin C metabolites catalyzed by NADPH-cytochrome P-450 reductase and xanthine oxidase. *J. Biol. Chem.*, **259**, 959–966.

PHILLIPS RM, HULBERT PB, BIBBY MC, SLEIGH NR AND DOUBLE JA. (1992). *In vitro* activity of the novel indoloquinone EO-9 and the influence of pH on cytotoxicity. *Br. J. Cancer*, **65**, 359–364.

ROBERTSON N, STRATFORD IJ, HOULBROOK S, CARMICHAEL J AND ADAMS GE. (1992). The sensitivity of human tumour cells to quinone bioreductive drugs: what role for DT-diaphorase? *Biochem. Pharmacol.*, **44**, 409–412.

ROSS D, SIEGEL D, BEALL H, PRAKASH AS, MULCAHY RT AND GIBSON NW. (1993). DT-diaphorase in activation and detoxification of quinones – bioreductive activation of mitomycin-C. *Cancer Metastasis Rev.*, **12**, 83–101.

SCHILTZ P AND KOHN H. (1992). Reductively activated mitomycin C: an efficient trapping reagent for electrophiles. *J. Am. Chem. Soc.*, **114**, 7958–7959.

SHARKIS DH AND SWENSON RP. (1989). Purification by Cibacron Blue F3GA dye affinity chromatography and comparison of NAD(P)H: quinone reductase (E.C.1.6.99.2) from rat liver cytosol and microsomes. *Biochem. Biophys. Res. Commun.*, **161**, 434–441.

SIEGEL D, BEALL H, SENEKOWITSCH C, KASAI M, ARAI H, GIBSON NW AND ROSS D. (1992). Bioreductive activation of mitomycin-C by DT-diaphorase. *Biochemistry*, **31**, 7879–7885.

WALTON MI, SMITH PJ AND WORKMAN P. (1991). The role of NAD(P)H-quinone reductase (EC 1.6.99.2, DT-diaphorase) in the reductive bioactivation of the novel indoloquinone antitumor agent EO9. *Cancer Commun.*, **3**, 199–206.

WALTON MI, BIBBY MC, DOUBLE JA, PLUMB JA AND WORKMAN P. (1992). DT-diaphorase activity correlates with sensitivity to the indoloquinone EO9 in mouse and human colon carcinomas. *Eur. J. Cancer*, **28A**, 1597–1600.

WORKMAN P. (1992). Keynote address – bioreductive mechanisms. *Int. J. Radiat. Oncol. Biol. Phys.*, **22**, 631–637.